# In Vitro and Biological Characterization of Dexamethasone Sodium Phosphate Laden pH-Sensitive and Mucoadhesive Hydroxy Propyl β-Cyclodextrin-g-poly(acrylic acid)/Gelatin Semi-Interpenetrating Networks

**DOI:** 10.3390/gels8050290

**Published:** 2022-05-07

**Authors:** Nyla Ajaz, Ikram Ullah Khan, Muhammad Irfan, Syed Haroon Khalid, Sajid Asghar, Yasir Mehmood, Muhammad Asif, Usra, Ghulam Hussain, Yasser Shahzad, Shefaat Ullah Shah, Muhammad Usman Munir

**Affiliations:** 1 Department of Pharmaceutics, Faculty of Pharmaceutical Sciences, Government College University Faisalabad, Faisalabad 38000, Pakistan; nylaajaz@gmail.com (N.A.); ikramglt@gmail.com (I.U.K.); manipharma1@gmail.com (M.I.); haroonkhalid80@gmail.com (S.H.K.); sajuhappa@gmail.com (S.A.); yasirmehmoodamjad@gmail.com (Y.M.); 2 Department of Pharmacology, Faculty of Pharmacy, The Islamia University of Bahawalpur, Bahawalpur 63100, Pakistan; drmasif@iub.edu.pk; 3 Department of Physiology, Government College University Faisalabad, Faisalabad 38000, Pakistan; usrakhan1990@gmail.com (U.); hussain806@gmail.com (G.H.); 4 Department of Pharmacy, COMSATS University Islamabad, Lahore Campus, Lahore 54700, Pakistan; yasi.shahzad@gmail.com; 5 Skin/Regenerative Medicine and Drug Delivery Research, GCPS, Faculty of Pharmacy, Gomal University, Dera Ismail Khan 29050, Pakistan; shefaatbu@gmail.com; 6 Department of Pharmaceutical Chemistry, College of Pharmacy, Jouf University, Sakaka 72388, Saudi Arabia

**Keywords:** semi-interpenetrating network, hydroxy propyl β cyclodextrin, gelatin, acrylic acid, dexamethasone

## Abstract

The current study reports the fabrication and biological evaluation of hydroxy propyl β-cyclodextrin-g-poly(acrylic acid)/gelatin (HP-β-CD-g-poly(AA)/gelatin) semi-interpenetrating networks (semi-IPN) for colonic delivery of dexamethasone sodium phosphate (DSP). The prepared hydrogels showed pH-dependent swelling and mucoadhesive properties. The mucoadhesive strength of hydrogels increased with an increasing concentration of gelatin. Based on the swelling and mucoadhesive properties, AG-1 was chosen as the optimized formulation (0.33% *w/w* of gelatin and 16.66% *w/w* of AA) for further analysis. FTIR revealed the successful development of a polymeric network without any interaction with DSP. SEM images revealed a slightly rough surface after drug loading. Drug distribution at the molecular level was confirmed by XRD. In vitro drug release assay showed pH-dependent release, i.e., a minute amount of DSP was released at a pH of 1.2 while 90.58% was released over 72 h at pH 7.4. The optimized formulation did not show any toxic effects on a rabbit’s vital organs and was also hemocompatible, thus confirming the biocompatible nature of the hydrogel. Conclusively, the prepared semi-IPN hydrogel possessed the necessary features, which can be exploited for the colonic delivery of DSP.

## 1. Introduction

Polymeric drug delivery systems are playing an important role in the safe and effective delivery of active pharmaceutical agents. Multicomponent polymeric delivery systems give opportunities to tailor the properties of carriers by manipulating the proportion of individual components. For instance, hydrogels are three-dimensional polymeric networks, which have the ability to retain a large portion of water. These homo or copolymer networks are often weak and gave a premature release of encapsulated drugs. Such limitations are easily addressed by interpenetrating polymeric networks (IPNs), which consist of two or more independent polymer networks and are prepared in the immediate presence of one another [1,2]. Various properties of IPN hydrogels such as strength, porosity, adhesion, degree of swelling, hydrophilicity, biocompatibility, loading, and drug release properties are conveniently controlled by a careful selection of components [3,4]. For example, dually cross-linked hydrogels were found suitable for wound healing [5], improved network properties, facilitated the incorporation and release of hydrophobic and hydrophilic drugs [6,7] and, moreover, chitosan-based (AMPS-co-AA) semi-IPN hydrogels were pH-sensitive and non-cytotoxic [8]. In the case of semi-IPNs, one polymer develops a cross-linked network, while the second polymer is physically entrapped as a linear or in branched form. So far, various natural and synthetic polymers have been investigated for the development of semi-IPN hydrogels to deliver various active constituents.

Cyclodextrin (CDs) are cyclic oligosaccharides containing different dextrose units (i) α(6), (ii) β(7), and (iii) γ(8) [9]. Among all CDs, HP-β-CD is mostly preferred, due to its ability to enhance solubility, improve bioavailability, increase drug loading, and maximize the stability of entrapped moiety [10]. Gelatin is an ionic, hydrophilic macromolecule derived from the hydrolysis of collagen. It is a biodegradable and biocompatible natural polymer [11] with swelling, gelling, bioadhesive, and film-forming ability, thus making it ideal for the development of biocompatible hydrogels [12]. It has the capacity to absorb ten times its weight of water [13]. Amino (–NH_2_) and carboxyl (–COOH) groups in gelatin help in cross-linking, enhance hydrophilicity, ease of functionalization, and impart gel strength to hydrogels [14,15]. These ionizing groups swell at low and high pH, so they can be used for making sustained release drug delivery systems [13]. Pure gelatins have poor mechanical strength, short degradation time, and it is difficult to shape them into hydrogels. This limitation can be overwhelmed by cross-linking or caging them in another network, as in semi-IPN [16]. In its cross-linked form, gelatin can make highly swellable complexes [17]. Moreover, it has mucoadhesive properties, which are used for diverse applications [18,19,20,21,22]. Acrylic acid is a well-known monomer that imparts pH-sensitive properties to developed networks [23,24,25]. These ingredients can be effectively combined to develop colon-specific carriers. Apart from pH-sensitive carriers, other approaches to achieving colon targeting include mucoadhesive carriers, covalent linkage of a drug with a carrier, timed released systems, carriers degraded specifically by colonic bacteria, and osmotic controlled drug delivery systems to various local diseases such as ulcerative colitis, Crohn’s disease, irritable bowel syndrome and colonic cancer [26,27,28]. The system will be more effective if it carries dual characteristics for targeting. For example, chitosan-based (AMPS-co-AA) semi-IPN hydrogels are pH-sensitive [8], while our proposed system carries both pH and mucoadhesive properties for targeting.

In the literature, the mucoadhesive and pH-sensitive behavior of hydrogel are reported by combining methacrylated gelatin and AA [29]. In another study, HP-β-CD was combined with carbopol 980 to prepare pH-induced mucoadhesive hydrogels for ocular delivery of dexamethasone [30]. Apart from the above studies, other studies mentioned these components in various combinations such as AA/gelatin hydrogels [31], gelatin-g-PVP-AA hydrogels [32], IPN hydrogels of gelatin and poly vinyl alcohol (PVA) [33], PVP, IA, and gelatin-based IPN hydrogels [34].

In the current research, gelatin was used as an interpenetrating polymer with mucoadhesive properties. AA imparted pH sensitivity to the network while drug loading will be improved by HP-β-CD. To the best of our knowledge, this combination is not yet reported. Developed mucoadhesive and pH-sensitive hydrogels will be extensively tested by various in vitro and in vivo techniques.

## 2. Methodology

### 2.1. Chemicals

Acrylic Acid (AA), gelatin (Type B, CAS No 9000-70-8) and ammonium persulphate (APS) were acquired from Daejung reagents chemicals and metals Co, Ltd., Nakdong-daero, Sasang-gu, Busan, Korea. Hydroxyl propyl β cyclodextrin (HP-β-CD) was received as a kind gift from Roquette, Lestrem, France. *N*,*N*-methylene-bis-acrylamide (MBA) and ethanol were obtained from Alfa Aesar (Heysham, UK) and Merck (Darmstadt, Germany), respectively. Dexamethasone sodium phosphate (DSP) was donated by Remington Pharmaceutical Industries, Lahore, Pakistan. All other chemicals used were of analytical grade.

### 2.2. Synthesis of HP-β-CD-g-poly(AA)/Gelatin Semi-IPN Hydrogels

**Figure 1:**
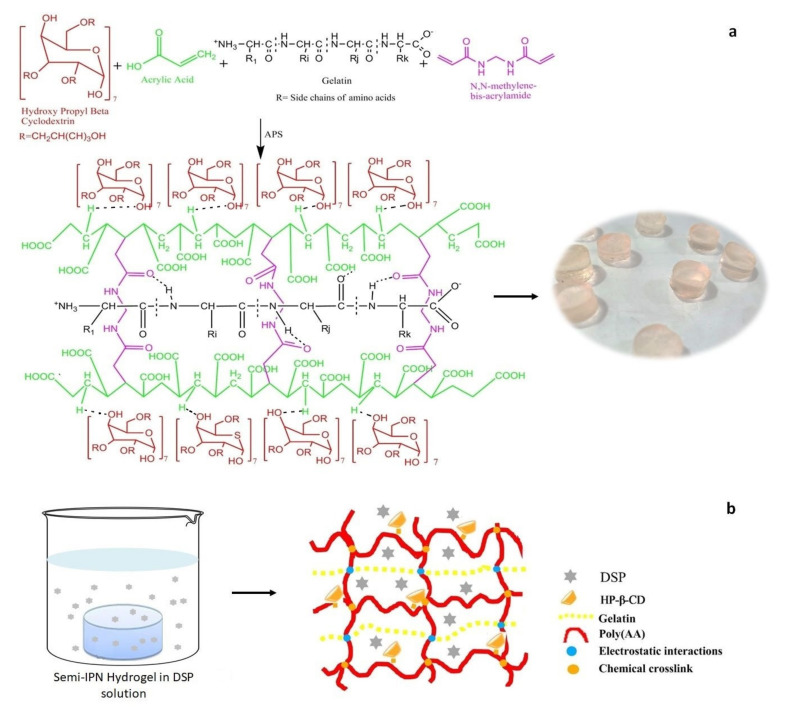
(**a**) Chemical structures of polymers, monomer, and cross-linker. Possible cross-linked structure and appearance of DSP-loaded HP-β-CD-g-poly(AA)/gelatin semi-IPN hydrogels. (**b**) DSP loading in semi-IPN hydrogel.

Semi-IPN hydrogels were synthesized by free radical polymerization with minor modification as reported previously [35]. Briefly, polymeric (gelatin and HP-β-CD) solutions were prepared independently and later mixed to get a homogenous solution (solution I). Initiator (APS) solution was separately prepared and added dropwise into specified amount of AA (solution II). Then, MBA solution was added dropwise into solution II with continuous stirring to get solution III. Afterward, polymeric solution was added dropwise to solution III under constant stirring. Finally, resultant solution was purged with nitrogen for 30 min. The resultant reaction mixture was poured into glass test tubes and placed in temperature-controlled water bath at 45 °C for 1 h. After that, temperature was changed to 50 °C for 2 h, then 55 °C for 3 h, 60 °C for 15 h, and finally 65 °C for 3 h. Cylindrical hydrogels were carefully removed from test tubes and cut into 6 mm discs. All the discs were washed with water and ethanol mixture (50:50 *v*/*v*) to remove unreacted or non-crosslinked monomers. These discs were dried in pre-heated oven at 45 °C for 24 h and then stored in airtight containers until further analysis. Fifteen formulations were developed by using components specified in Table 1. The proposed reaction scheme is presented in Figure 1.

### 2.3. Swelling Study

To investigate the pH-dependent swelling behavior, the dried, pre-weighted hydrogel discs were immersed in buffer solution of pH 1.2 and 7.4. The swelling data were obtained by weighing the discs at different time intervals until equilibrium. The swelling ratio (SR) was calculated using Equation (1) [36,37].

SR (g/g) = W_t_/W_0_ (1)

where W_t_ = weight of swollen semi-IPN hydrogel; W_0_ = weight of dried semi-IPN hydrogel before swelling.

### 2.4. Mucoadhesive Strength

The mucoadhesive properties of DSP-loaded HP-β-CD-g-poly(AA)/gelatin semi-IPN hydrogels were evaluated by slight modification of previously reported method [38]. Here, the maximum weight required to detach the hydrogel from membrane was assessed. All experiments were carried out on freshly excised rabbit intestine, which was washed in normal saline (0.9% NaCl *w*/*v*) and soaked in PBS of pH 7.4 at 37 °C for 30 min. Afterward, hydrogel discs were placed on membranes by applying constant weight of 5 g for 1 min. Mucoadhesive strength was measured in terms of force per unit area.

### 2.5. Sol–Gel Analysis

Sol–gel fraction analysis was performed by previously reported method [39]. Hydrogel discs of 6 mm were dried in oven at 45 °C to constant weight. Afterward, all discs were exposed to distilled water for 48 h to remove non-crosslinked fraction. After 48 h, hydrogel discs were removed from distilled water and dried in oven at 45 °C to constant weight. Sol and gel fraction of respective formulation was calculated by using the following equations [15]:

(2) $$\mathrm{Gel}\;\mathrm{Fraction}\;=\;\frac{Wf}{Wi}\times 100$$

Sol Fraction = 100 − Gel Fraction(3)

where W_f_ = final weight of extracted dried semi-IPN hydrogel, W_i_ = initial weight of dried semi-IPN hydrogels.

### 2.6. Solid-State Characterization

FTIR spectra of pure drug, blank, and drug-loaded HP-β-CD-g-poly(AA)/gelatin semi-IPN hydrogels were obtained in the range of 4000–500 cm^−1^. XRD diffraction patterns of pure drug, blank, and drug-loaded semi-IPN hydrogels were obtained on X’pert PRO, PANalytical, Almelo, The Netherlands. SEM (FEI Quanta 250, Hillsboro, OR, USA) was used to evaluate the surface morphology of drugs and hydrogels.

### 2.7. Drug Loading

Semi-IPN hydrogel discs (AG-1) were weighed and allowed to swell in drug solution at 37 °C for 48 h 1% *w*/*w* DSP was prepared in PBS of pH 7.4. Subsequently, hydrogel discs were removed from the drug solution and blotted with filter paper to remove excess water. Finally, dried in oven at 45 °C until constant weight. The following equations were used to calculate amount of drug load in hydrogels [13]:

Amount of drug = W_D_ − W_d_ (4)

Percentage drug loading was calculated by the following equation:

(5) $$\mathrm{Drug}\;\mathrm{Loading}\;(\%)\;=\;\left[\frac{W_{D}-W_{d}}{W_{d}}\right]\times 100$$

where W_D_ = weight of dried semi-IPN hydrogels after loading, W_d_ = weight of dried semi-IPN hydrogels before loading.

### 2.8. In Vitro Drug Release

In vitro drug release profile of semi-IPN hydrogel (AG-1) was obtained by using a dissolution apparatus II (Tianjin Guoming Medicinal Equipment Co. Ltd., Tianjin, China) at 37 °C ± 0.5 and 50 rpm. Initially, drug release was studied in 500 mL of HCl buffer (pH 1.2) for 2 h. Afterward, discs were transferred to 500 mL of PBS (pH 7.4) for next 70 h. For analysis, 5 mL of sample was collected at specific intervals and replaced with fresh release medium. These samples were passed through 0.45 μm syringe filter and analyzed by UV Visible spectrophotometer (Agilent, Santa Clara, CA, USA, Model 8453) at 242 nm. Cumulative percent release (%) was calculated using the following equation [40]. Experiment was performed in triplicate and average percent drug release was taken to draw the release curves.

(6) $$\%\;\mathrm{drug}\;\mathrm{release}\;=\;\frac{M_{t}}{M_{n}}\times 100$$

where M_t_ shows the quantity of DSP released at any time “t” and M_n_ represents the quantity of DSP loaded in AG-1. After obtaining DSP release data, the Korsmeyer–Peppas model was used to elucidate drug release mechanism as follows [39]:

M_t_/M_o_ = kt^n^ (7)

where M_t_/M_o_ = amount of drug released at time “t” and infinity, k = release rate constant for Korsmeyer–Peppas model, and n = diffusion coefficient.

### 2.9. Hemocompatibility Testing

Hemocompatibility test was performed on optimized formulation (AG-1) according to the previously reported method [41]. The test was conducted according to guidelines of International Standard Organization (ISO) and approved by Ethical Committee (ERC) of GCUF (Reference no. GCUF/ERC/2153). Human blood samples were collected from healthy donors in 4.5 mL tubes containing 3.2% sodium citrate. Blood samples were used within 1 h after collection. Blood was incubated with blank and drug-loaded hydrogels: 100 mg of each hydrogel was exposed to 900 μL of blood for 15 min at 37 °C. After incubation, 5 μL of blood was dropped on a glass slide, spread immediately, and observed under microscope (Accu-Scope 3001 Trinocular, Commack, NY, USA) fitted with 5-megapixel camera at 40× to study compatibility of hydrogels with human blood cells. Moreover, 100 μL of blood samples were taken in micro Eppendorf tubes for biochemical analysis.

For analysis of hemolysis ratio (HR), 2 mL of fresh human blood with anticoagulant was added to the hydrogel sample with addition of 5 mL PBS (7.4 pH) and considered as test sample. Positive and negative controls were prepared by adding same amount of blood to distilled water and PBS, respectively. Then these tubes were incubated for 60 min at 37 °C followed by centrifugation at 2500 rpm for 20 min. The absorbance of supernatant was observed at 575 nm by UV Visible spectrophotometer (Agilent, Santa Clara, CA, USA, Model 8453). HR was calculated by the following equation [42]:

(8) $$\mathrm{HR}\;(\%)\;=\;\frac{A_{S}\;-\;A_{\mathrm{NC}}}{A_{\mathrm{PC}}\;-\;A_{\mathrm{NC}}}\;\times \;100$$

where A_S_ is the absorbance of sample, A_NC_ is the absorbance of the negative control, and A_PC_ is the absorbance of the positive control.

### 2.10. Toxicity Testing

Acute toxicity of optimized formulation (AG-1) was performed according to fixed-dose guideline no. 420 as given by the Organization for Economic Co-Operation and Development (OECD). All protocols were approved by ERC of GCUF via letter no. GCUF/ERC/2026. In brief, healthy rabbits were housed in clean cages for acclimatization. Rabbits were randomly divided into two groups, each with three rabbits. Control group was administered with food and water only, while treated group received 2 g/kg of DSP-loaded HP-β-CD-g-poly(AA)/gelatin.

After that, animals were continuously observed for behavioral patterns, mortality rate, body weight, food, and water consumption for fourteen days. At the end of study, blood samples were taken for biochemical and hematological analysis. Finally, animals were sacrificed to remove vital organs (heart, kidney, liver, lung, and stomach) and dipped in formalin solution (10% *v*/*v*). Slides were developed and used for histopathological analysis.

### 2.11. Statistical Analysis

Statistical analysis of data was executed by Graph Pad Prism 5.0. One-way ANOVA and Student’s *t*-test were applied according to the number of groups studied. For *p*-value < 0.05 was considered a significant difference in data.

## 3. Results and Discussion

### 3.1. Swelling Studies

Swelling behavior and structural stability of hydrogels are two important parameters determining their use in controlled drug delivery. The degree of swelling directly influences the rate of water absorption, mechanical strength, and permeability of encapsulated drugs in hydrogels. Here, HP-β-CD, APS, and MBA were kept constant so the effect of other formulation factors (pH, AA, gelatin) could be studied.

#### 3.1.1. Effect of pH

**Figure 2:**
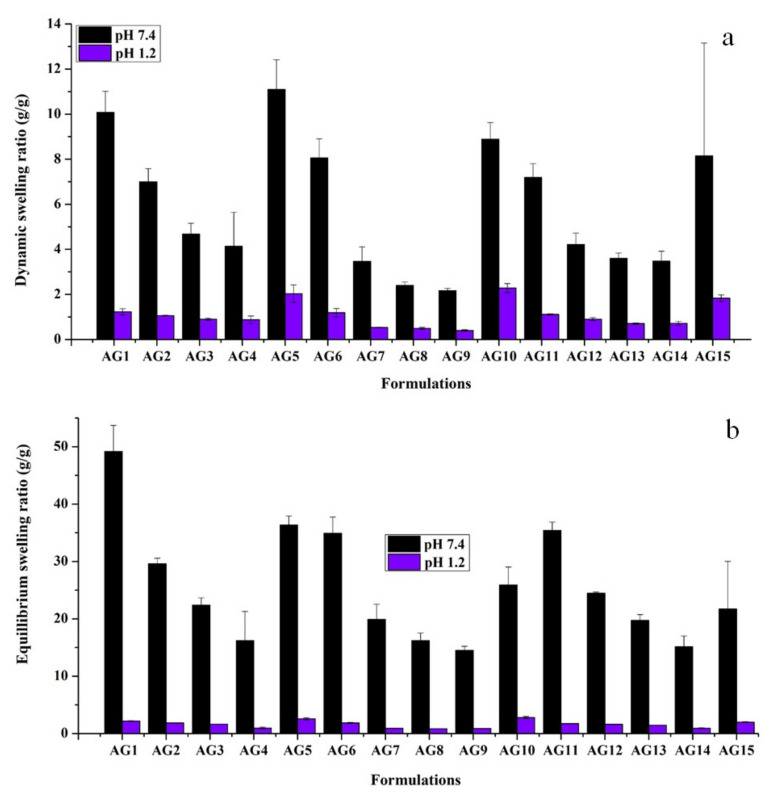
(**a**) Dynamic and (**b**) equilibrium SR of semi-IPN hydrogels. Dynamic SR was calculated at 8th hour. Error bar represents standard deviation. Here, data are presented as means ± SD (*n* = 3).

Two factors directly influence the swelling properties of the semi-IPN hydrogels: (i) the pH of the surrounding medium, and (ii) the pk_a_ value of acidic or basic groups of the polymer. Therefore, the prepared HP-β-CD-g-poly(AA)/gelatin networks were subjected to swelling studies at different pH levels to estimate their pH sensitivity. For all formulations, percent dynamic and equilibrium swelling was higher at a pH of 7.4 and lower at a pH of 1.2 (Figure 2). This variation affects the swelling of the hydrogels and can be linked with (i) AA, which promotes the swelling of hydrogels in basic media but collapses in acidic conditions. Moreover, the pk_a_ value of AA is 4.28 [12]. When the pH of the media is above the pk_a_ value of AA, –COOH groups are ionized to impart a net negative charge on the polymer chains, which leads to repulsion among hydrogel chains, and therefore swelling is increased at a pH of 7.4 [43]; (ii) Gelatin contains hydrophilic groups such as –NH_2_ and –COOH that also contribute towards swelling. In the literature, [44] fabricated β-CD/carboxy methyl cellulose-co-poly(AA) hydrogels and found similar swelling behavior at acidic and basic pHs.

#### 3.1.2. Effect of AA

**Figure 3:**
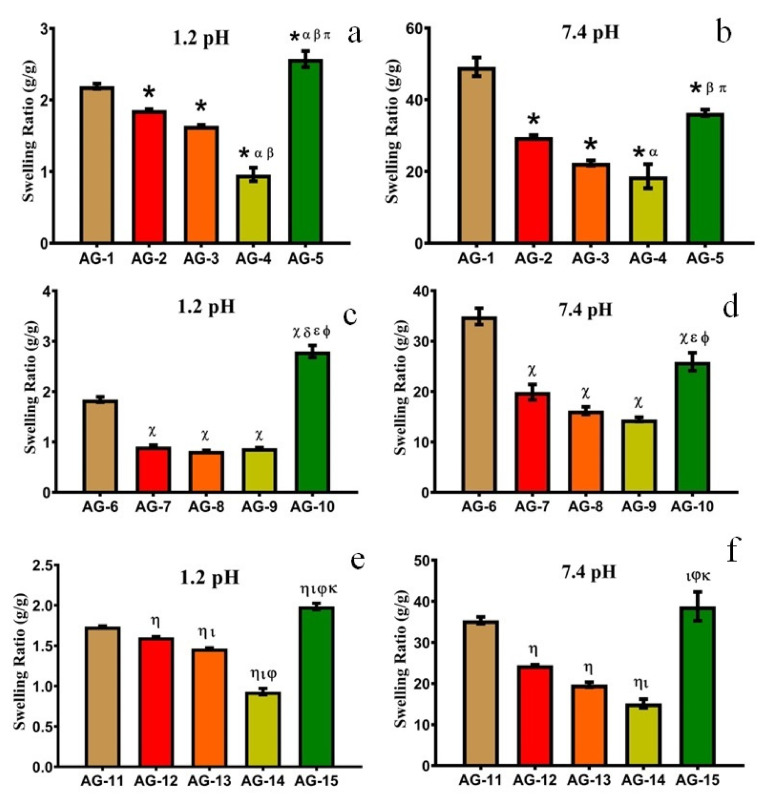
Effect of increasing AA contents on equilibrium SR at pH (**a**) 1.2 and (**b**) 7.4 with constant gelatin (0.33 g). pH (**c**) 1.2 and (**d**) 7.4 with constant gelatin (1.33 g). pH (**e**) 1.2 and (**f**) 7.4, with constant gelatin (2.66 g). All the data are presented as means ± SD (*n* = 3). * compared with AG-1, α compared with AG-2. β compared with AG-3, π compared with AG-4, χ compared with AG-6, δ compared with AG-7, ε compared with AG-8, ϕ compared with AG-9, η compared with AG-11, ι compared with AG-12, φ compared with AG-13, κ compared with AG-14.

AA is a well-known pH-sensitive monomer and its concentration has a marked influence on SR. When AA-based hydrogels are in contact with fluids of suitable pH, their networks swell and form porous structures. Such porous networks are altered by increasing or decreasing the cross-linking density. One can alter the network density of hydrogels by increasing or decreasing the concentration of AA and thus the release of the entrapped molecule can be controlled. It is observed that when AA contents are increased from 16.66 to 66.66 wt%, SR was decreased, as shown in Figure 3. This trend can be interpreted by the fact that AA is a small molecule. At low concentrations, it develops a loose network with enough spaces for easy access to the surrounding dissolution media, while at high concentrations, AA tends to develop a close and compact polymeric structure. This restricts the media movement and thus decreases the swelling of the prepared hydrogels. Similar results were obtained by [45], who found that 2.5 and 10% *w*/*w* of p(AA) in hydrogels showed 0.87 and 0.73 average swelling power, respectively. Moreover, these AA-based hydrogels showed higher swelling at pH 7.4 as compared to pH 1.2, which is because AA is more responsive to basic pH as compared to the acidic pH as discussed in detail in the previous section.

When the AA concentration was increased to the maximum, i.e., 83.33 wt%, SR increased all of a sudden, as shown in Figure 3. The probable reason is the development of low molecular weight polymers, which compromised gel strength and leads to rapid swelling in the given media [46]. Another probable reason is a decrease in cross-linking density with a higher concentration of acrylic acid at a constant concentration of crosslinker and polymers. Hydrogels with maximum AA (83.33 wt%) were mechanically weak, showed a sponge-like appearance, and were easily broken after swelling.

#### 3.1.3. Effect of Gelatin

**Figure 4:**
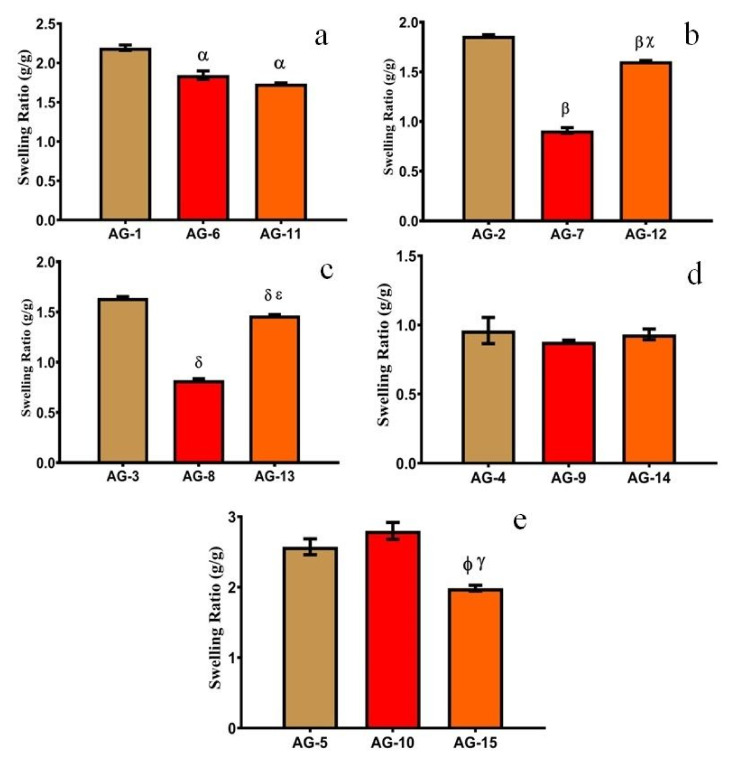
Effect of gelatin on equilibrium SR at pH 1.2 with different AA concentrations, i.e., (**a**) 16.66 g, (**b**) 33.33 g, (**c**) 50 g, (**d**) 66.66 g and (**e**) 83.33 g. All the data are presented as means ± SD (*n* = 3). *p* < 0.05, α compared with AG-1, β compared with AG-2, χ compared with AG-7, δ compared with AG-3, ε compared with AG-8, ϕ compared with AG-5, γ compared with AG-10.

**Figure 5:**
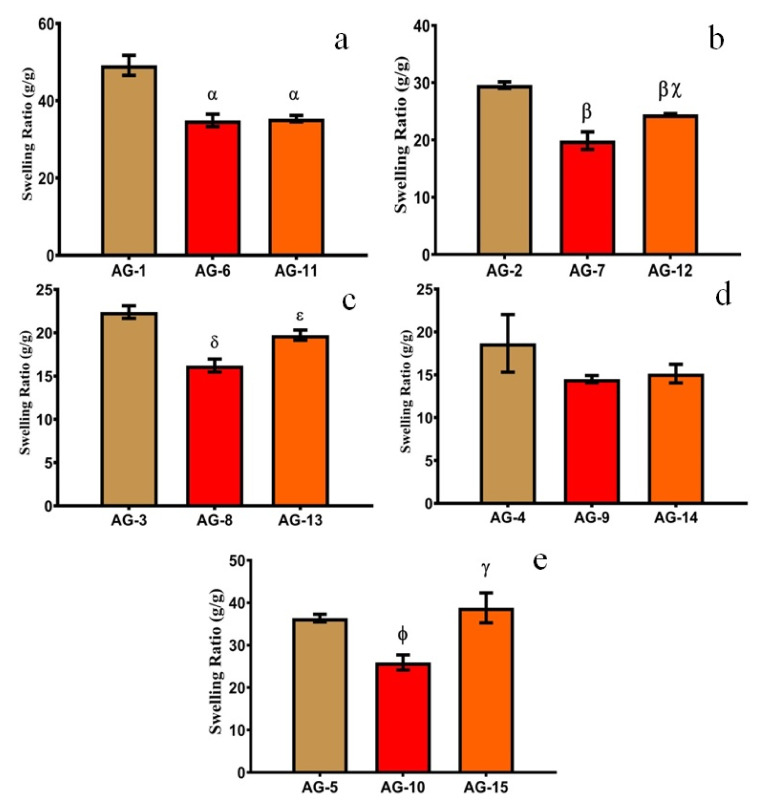
Effect of gelatin on equilibrium SR at pH 7.4 with different AA concentrations, i.e., (**a**) 16.66 g, (**b**) 33.33 g, (**c**) 50 g, (**d**) 66.66 g and (**e**) 83.33 g. Error bars presented as means ± SD (*n* = 3). *p* < 0.05, α compared with AG-1, β compared with AG-2, χ compared with AG-7, δ compared with AG-3, ε compared with AG-8, ϕ compared with AG-5, γ compared with AG-10.

In order to estimate the influence of gelatin concentration on SR, different formulations were developed with varying concentrations of gelatin (0.33, 1.33, and 2.66 wt%). They were exposed to different pH mediums (1.2 and 7.4), as displayed in Figure 4 and Figure 5, respectively.

The swelling of the prepared semi-IPN hydrogels decreased with the increasing contents of gelatin up to 1.33 wt%. A possible explanation for this behavior is (i) as the gelatin concentration is increased, the cross-link density is also increased, which leads to a decrease in swelling; (ii) At higher concentrations, gelatin occupies the interstitial spaces in a semi-IPN structure and leads to decreased swelling, as observed in previous studies [39]; (iii) With increasing gelatin concentration, the viscosity of the system also increases which decreases the mobility of chains. This limits the interaction with the dissolution media, which leads to a further decrease in swelling [47].

**Figure 6:**
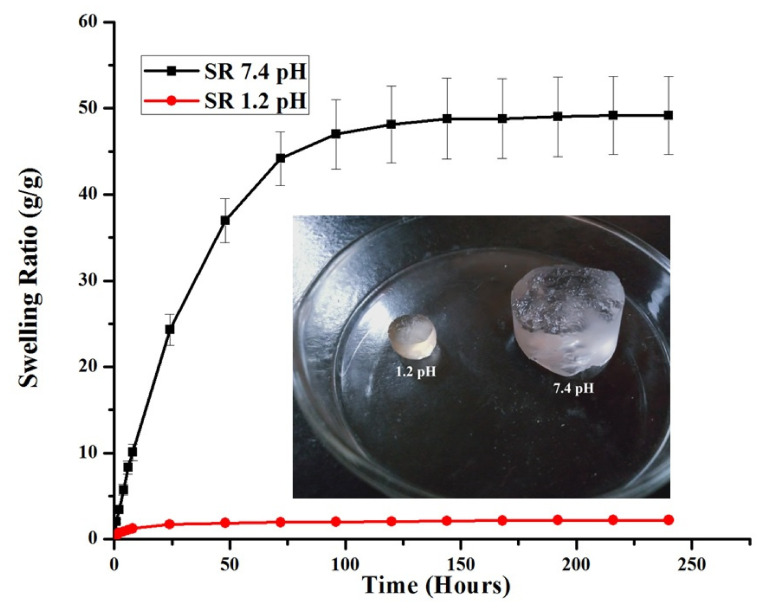
Equilibrium SR and physical appearance of AG-1 at pH 1.2 (red) and 7.4 (black). Data are presented as means ± SD (*n* = 3).

When the gelatin concentration was further increased from 1.33–2.66 wt%, the swelling of the hydrogels increases abruptly owing to a higher number of ionizable (–NH_2_ and –COOH) groups. This leads to higher hydrostatic pressure and the swelling increases abruptly [13]. A similar swelling pattern was observed by [48], who prepared cross-linked hydrogels of pectin. They found that with increasing contents of gelatin from 10% to 30%, the swelling ratio decreased from 324% to 195%. Moreover, with a further increase in gelatin contents (40%), swelling increased up to 240%. Thus, we can conclude that a low concentration of gelatin in semi-IPN hydrogels is necessary to achieve optimum swelling. SR and the physical appearance of AG-1 are shown in Figure 6.

### 3.2. Mucoadhesive Strength

**Figure 7:**
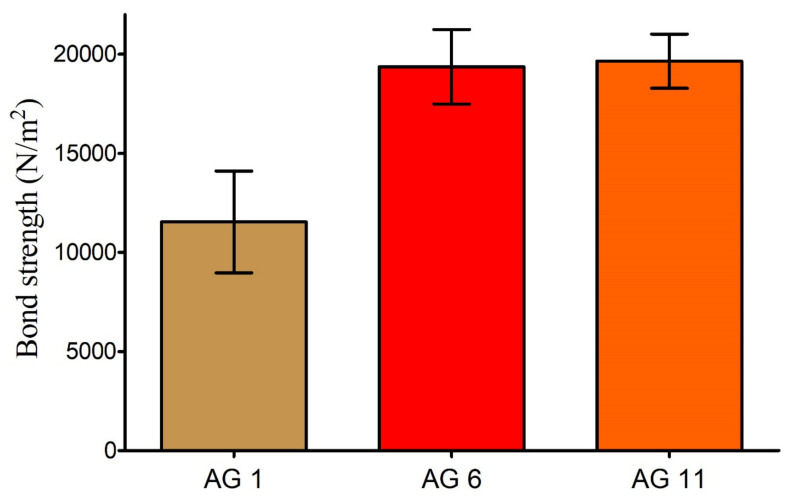
Mucoadhesive strength of prepared hydrogels with increasing concentration of gelatin (AG-1, AG-6, and AG-11). Error bars presents means ± SD (*n* = 3).

Mucoadhesion plays an important role in prolonging the contact of drug carriers with membranes and thus helps to enhance drug concentration at the site of action with improved therapeutic efficacy [49,50]. The mucoadhesive force of carriers is governed by nature and the concentration of mucoadhesive polymers [51]. Gelatin is a natural protein derived from partially denatured collagen and is reported to be biodegradable, biocompatible, non-immunogenic, non-toxic, and develops mucoadhesion by the hydration mechanism, where carboxyl groups of polymer interact with mucin [39,52]. Figure 7 shows increased mucoadhesion with the increasing concentration of gelatin with the highest bonding strength for AG-11. Similar results were reported by [29], who developed mucoadhesive and pH-responsive hydrogels from AA and methacrylated gelatin.

Although AG-11 showed the highest bond strength, we selected AG-1 as the optimized formulation as it was developed with the minimum possible formulation ingredients and showed superior swelling characteristics (discussed in the previous section). Furthermore, it is reported that excessive mucoadhesion could damage or irritate mucosal membranes and thus lead to patient noncompliance [51].

### 3.3. Sol–Gel Fraction

Sol–gel fraction was used to estimate the non-crosslinked portion of the prepared formulations. Here, gel (%) was directly affected, while sol (%) was inversely affected with increasing monomer contents in the HP-β-CD-g-poly(AA)/gelatin networks. At the lowest AA concentration (AG-1), 90% gelation was observed. Gel fraction increased while sol fraction decreased with an increasing concentration of AA, as shown in Table 2. Many researchers have reported a similar trend mentioning the increase in gel fraction (%) with an increasing concentration of monomer (AA) [31,53,54]. Although the gel (%) of AG-1 was on the lower side considering swelling and mucoadhesive results, AG-1 was chosen as an optimized formulation and considered for the next analysis.

### 3.4. Solid-State Characterization

Solid-state characterization has attained a central position in the pharmaceutical industry and drug development process. These techniques provide a sound basis for the establishment of a stable and efficacious formulation. The various techniques used to characterize the optimized hydrogels are discussed below:

#### 3.4.1. FTIR

FTIR provides useful information about functional groups that can be employed to elucidate the semi-IPN structure and rule out any possible interaction between the carrier and loaded drug.

The literature mentions numerous peaks of AA due to its functional groups. For example, (i) the –OH (hydroxyl group) peak is located at 3380 cm^−1^, (ii) the –CH_2_ (methylene group) peak is located at 2973 cm^−1^, (iii) the –C=O (carbonyl group) peak is located at 1750 cm^−1^ and 1718 cm^−1^, (iv) the –C–C group peak at 1709 cm^−1^, (v) the –C=C group peak is presented at 1637 cm^−1^, and (vi) the –C–O–C group peak is located at 1173 cm^−1^ [23,55,56,57,58]. Gelatin shows corresponding peaks of –NH stretching, –C=O, and –CH groups at 3416, 1639, 3060, and 2948 cm^−1^, while peaks at 1651 and 1564 cm^−1^ depicted amide I and II absorption bands. The peak at 1230 cm^−1^ may represent –CN bond vibration [31,59,60,61,62]. HP-β-CD shows peaks of –OH, –CH, and –C–O group at 3414, 2995, and 1158 cm^−1^, respectively [63].

**Figure 8:**
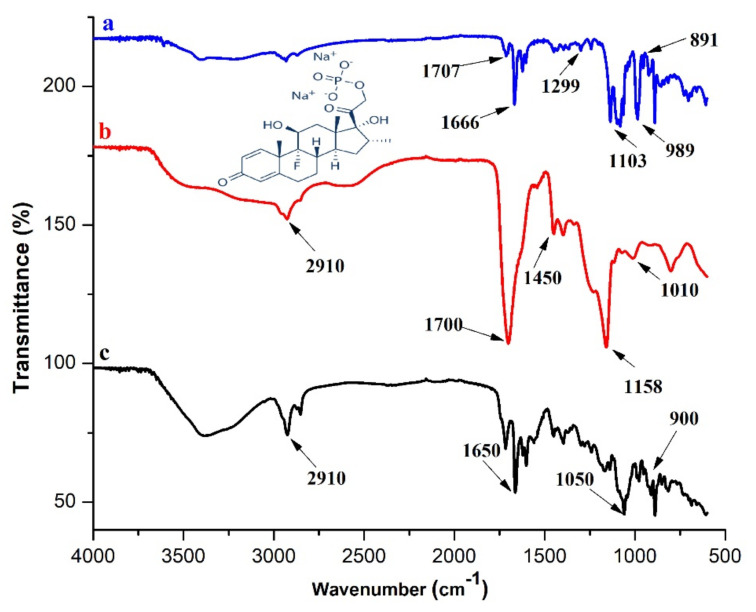
FTIR spectrum of: (**a**) DSP, (**b**) blank, and (**c**) loaded AG-1.

On observing the spectra of the blank HP-β-CD-g-poly (AA)/gelatin semi-IPN hydrogels (Figure 8b), we observed that absorption peaks disappeared or weakened between 3500–3000 cm^−1^. A strong absorption band at 3414 cm^−1^, which corresponds to the hydroxyl group of HP-β-CD disappeared. This confers the involvement of –OH groups of HP-β-CD in the polymerization reaction. Blank hydrogel spectra show two new peaks at 2910 and 1158 cm^−1^, which may represent –CH groups peaks of gelatin and –C–N groups of gelatin/or –C–O groups peaks of HP-β-CD, respectively. In blank hydrogels, a very small absorption peak is depicted at 1010 cm^−1^, which may represent the stretching of –C–O–C groups of HP-β-CD. Blank gels showed a –C=C group peak of AA that shows unsaturated bonds of AA have successfully reacted with other reactants. Similar observations were reported by [64], who prepared gelatin-g-poly(acrylic acid-*co*-acrylamide).

FTIR spectra of the pure drug (Figure 8a) show characteristic absorption peaks at 1707, 1666, and 1624 cm^−1^ that are analogous to –C=O bonds in the drug [65]. Notably, vibrational peaks at 1299 and 1103 cm^−1^ may be due to the phosphate anion (–PO) of DSP [66]. The –CF group axial deformation of DSP shows very small peaks at 989 and 891 cm^−1^ [67]. Moreover, blank and drug-loaded spectra of HP-β-CD-g-poly(AA)/gelatin hydrogels are almost similar. In the DSP-loaded spectra of the semi-IPN hydrogel (Figure 8c), a new peak appears at 1050 cm^−1^, which may be due to the merging of peaks of –PO of DSP. The –CF group peaks (989 cm^−1^, 891 cm^−1^) assimilate at 900 cm^−1^ thus indicating the presence of DSP in the IPN hydrogels.

#### 3.4.2. XRD

**Figure 9:**
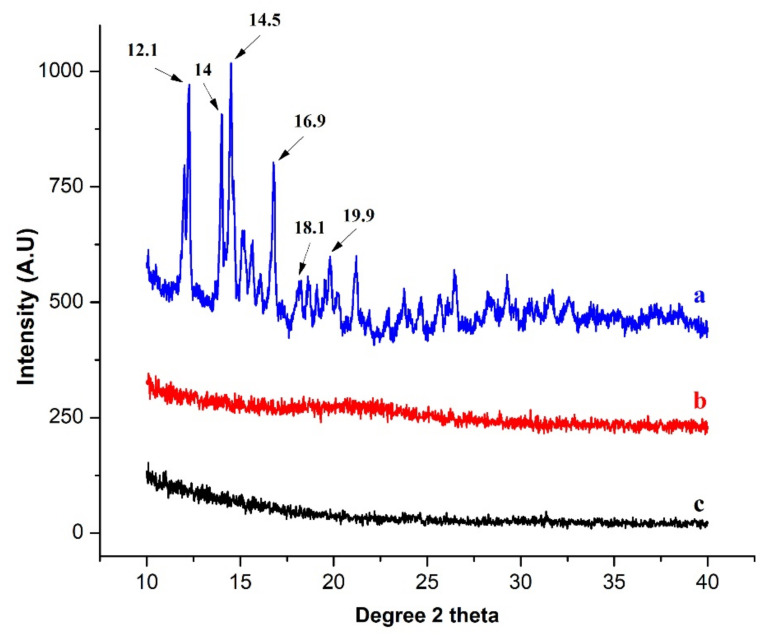
XRD histogram of: (**a**) DSP, (**b**) blank, and (**c**) drug-loaded AG-1 semi-IPN hydrogels.

XRD provides useful information about the basic structure and nature of the material (crystalline or amorphous). The XRD spectra of pure drug, blank, and loaded hydrogels are presented in Figure 9.

The crystalline nature of DSP was confirmed by sharp peaks between 12° and 25° (Figure 9), as reported previously [68]. The amorphous nature of the blank hydrogel was due to the amorphous nature of its constituents. For example, poly(AA), HP-β-CD (shows two wide peaks, i.e., (i) 5–15° and (ii) 15–25°) [63,69] and gelatin are amorphous in nature [70,71]. In blank hydrogel spectra, these peaks were further decreased. On the other hand, DSP-loaded spectra also suggest complete dispersion of the drug at the molecular level, or their peaks are camouflaged by the polymeric network. In one study, [12] fabricated hydrogels by using gelatin, AA, and AMPS (2-acrylamido-2-methylpronesulfonic acid) and loaded oxaliplatin. No peaks of oxaliplatin were observed in the XRD spectra of the loaded hydrogel showing the dispersion of the drug at the molecular level.

#### 3.4.3. SEM

**Figure 10:**
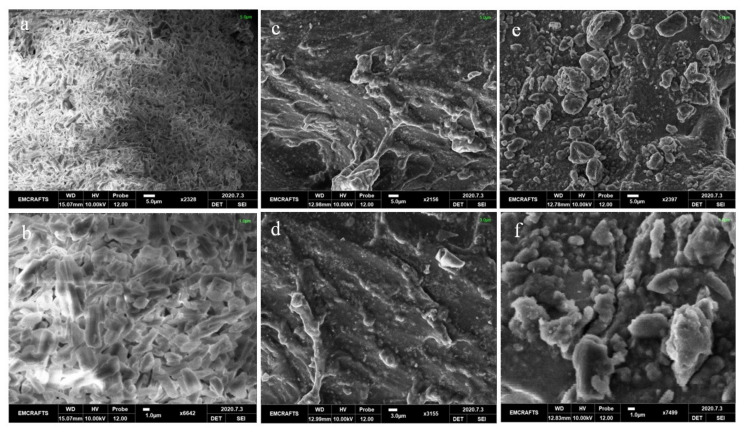
Surface morphology of DSP (**a**,**b**), blank (**c**,**d**) and loaded (**e**,**f**) semi-IPN hydrogels as observed under SEM.

SEM is widely used to study the surface morphology of semi-IPN hydrogels. SEM images show the crystals of pure DSP, as shown in Figure 10a,b, which show its crystalline nature [72]. This further supplements the XRD results of DSP.

Blank gels showed uneven, compact, and non-porous structures owing to the formation of cross-linked networks. The surface of the drug-loaded semi-IPN hydrogel (Figure 10e,f) was also non-porous but comparatively rough with particles dispersed throughout the surface, which gives a hint about the DSP loading in the hydrogel. These surface adhered particles may propose initial DSP release at pH 1.2 as will be discussed in the in vitro drug release study section. Sethi and colleagues prepared dialdehyde carboxy methyl cellulose–gelatin hydrogels and found a similar rough compact structure of cross-linked hydrogels [14].

### 3.5. DSP Loading and In Vitro Release Study

In this study, an optimized semi-IPN hydrogel (AG-1) containing 216.86 ± 29.56 mg of DSP was chosen for drug release studies. Here, we observed a marked improvement of drug loading after HP-β-CD grafting as compared to the previous study, where 170.54 ± 1.75 mg of DSP was loaded in a similar-sized disc of pectin-g-poly(AA)/PVP semi-IPN networks [73].

**Figure 11:**
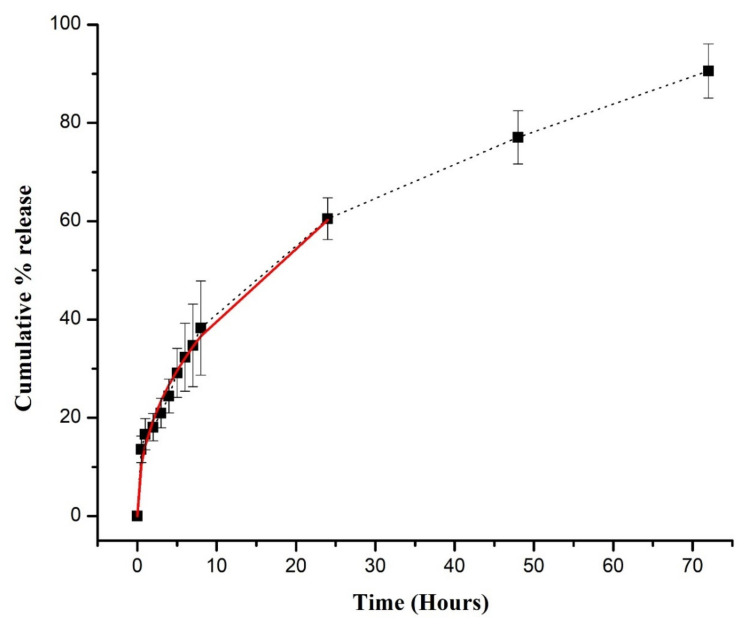
In vitro DSP release from semi-IPN hydrogels (AG-1) (shown by black dotted line). Release curve shows Korsmeyer–Peppas model on the first 60% release (shown by red continuous line). Error bars represents means ± SD (*n* = 3).

The pH of the release media significantly affects the drug release rate from the pH-sensitive semi-IPN hydrogels, therefore it was determined at two different pHs (1.2 and 7.4). For the initial 2 h, DSP release was carried out at low pH while for the next 70 h the release medium was changed to a higher pH. The DSP release rate from the hydrogels is directly related to their swelling rate. A small quantity of DSP was released (18.1%) at pH 1.2 (Figure 11) and can be explained by the following facts. (a) In acidic pH, hydrogels remain in a collapsed form due to intact –COOH groups and thus prevent the release of DSP. (b) Even in the collapsed form, a small amount of DSP was released, which is due to the surface adhered DSP, as revealed in SEM micrographs. At pH 7.4, the DSP release rate gradually increased with time, and approximately 90.58% of the entrapped drug was released at the end of the study. In an alkaline pH, hydrogels show enhanced swelling owing to ionization of the –COOH groups that leads to electrostatic repulsion between the chains of the semi-IPN network. Thus, a higher quantity of the drug was released at the desired pH. Consequently, these networks can enhance the therapeutic efficacy of DSP to treat ulcerative colitis and inflammatory bowel disease. Furthermore, they will also decrease DSP systemic side effects, which are usually observed with prolonged usage. The authors in [74] prepared and characterized pH-responsive IPN hydrogels of gelatin-poly(methacrylic acid) containing glipizide. They found fewer drug releases in the first 2 h at pH 1.2, while the release rate increased afterward at pH 7.4.

### 3.6. Drug Release Modeling

The Korsmeyer–Peppas model was applied to release data to gain insight into the mechanism of drug release from the hydrogels [75]. The correlation coefficient (R^2^) was near unity, indicating the suitability of the model. If the “*n*” value is less than 0.45, this represents Fickian diffusion; if 0.45 < *n* < 0.89, this represents non-Fickian diffusion and if the “*n*” value is greater than 0.89, this represents the case II transport mechanism. For AG-1, the value of the diffusion exponent was above 0.45 (Table 3), indicating a non-Fickian diffusion mechanism. Shah et al. developed chlorpheniramine maleate-loaded poly(AA)/poly vinyl alcohol IPNs. These gels released the loaded drug by non-Fickian diffusion [35].

### 3.7. Hemocompatibility Test

**Figure 12:**
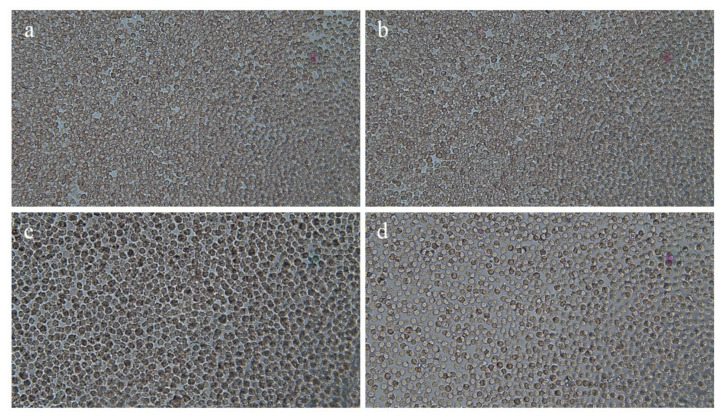
Human blood specimen exposed to hydrogels, (**a**) Control (**b**) Blood after incubation without hydrogel contact, (**c**) Blood in contact with blank gels (AG-1), (**d**) Blood in contact with DSP-loaded gels (AG-1). Photos were taken by using Trinocular (Accu-Scope 3001) microscope equipped with 5-megapixel camera at 40×.

The hemocompatibility test helps to assess the hemocompatible nature of the hydrogels. Figure 12a shows a control human blood specimen with no contact with the hydrogel. Then, a portion of blood was incubated (Figure 12b) without any contact with the hydrogel at 37 °C. Afterward, blood samples were incubated with blank (Figure 12c) and DSP-loaded hydrogels (Figure 12d). All samples presented normal-shaped blood cells, which pointed toward the biocompatible nature of gels. Similar findings were reported by [76] for hydrogel films of xyloglucan-poly vinyl alcohol. They observed that the shape of the erythrocytes was comparable to the control. Furthermore, biochemical analysis (Table 4) showed no difference in hemoglobin level, number of RBCs, WBCs, and the platelet count of control and treated groups, respectively.

**Figure 13:**
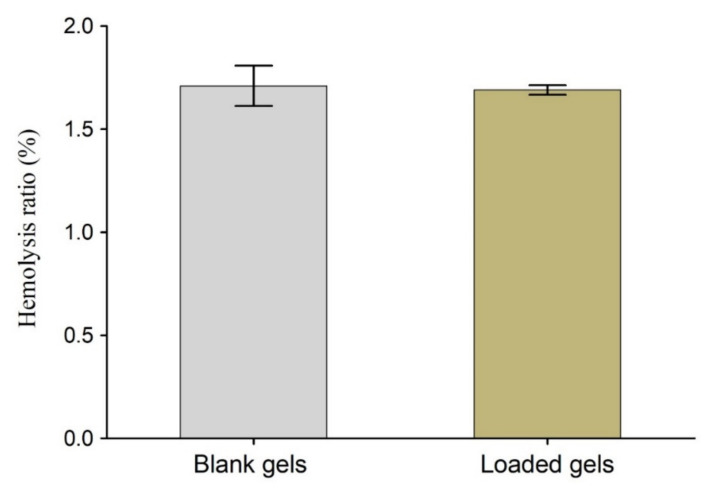
Hemocompatibility study of blank and drug-loaded semi-IPN hydrogels (AG-1). Error bars represents means ± SD (*n* = 3).

After microscopic and biochemical analysis of the blood, we determined the hemolysis ratio (HR). This determines the amount of hemoglobin (Hb) discharged from the red blood cells (RBCs) upon contact with the test material [77]. Thus, it gives more accurate and conclusive information about the hemocompatibility of the material under testing. If the HR of the hydrogel is below 2%, it is termed as non-hemolytic, between 2–5% as slightly hemolytic, and >5% as a hemolytic sample [78]. The HR (%) of the blank and drug-loaded hydrogels (AG-1) was less than 2% (Figure 13). Our results confirm the hemocompatible nature of the HP-β-CD-g-poly(AA)/gelatin semi-IPN hydrogels. Thus, this will not alter the integrity of the blood components if used in vivo. Similar findings were reported for fast swelling porous superabsorbent hydrogels of (starch-g-poly(AA-*co*-acrylamide)), where % hemolysis was <2%, thus indicating the non-hemolytic nature of the developed hydrogels [79].

### 3.8. Toxicity Testing

#### 3.8.1. Monitoring the General Conditions of the Rabbits

Single or multiple oral doses of any substance may cause toxic effects over a short period of time and are referred to as acute toxicity [62]. In this study, all rabbits behaved normally without any signs of toxicity, illness, or death. No major differences in water and food consumption were recorded before and after treatment, as shown in Table 5. During treatment, body weight changes are regarded as a key indicator of toxicity. If this decrease is 10% or more it will reflect the toxic nature of the carrier. In the current study, no significant weight changes were observed among the control and treated groups. The authors in [80] synthesized xanthan gum/PVP-*co*-poly-(AA) interpenetrating hydrogels and found no significant clinical changes between the control and treated groups. Similar observations were reported by [81], who prepared semi-IPN hydrogels based on β-CD and IA-co-AMPS and found no differences in weight and other clinical observations of animals in the control and treated groups.

#### 3.8.2. Biochemical and Hematological Observation

After oral treatment, no major differences in the biochemical and hematological parameters of control and drug-loaded HP-β-CD-g-poly(AA)/gelatin were observed, as evident from Table 6 and Table 7. Hence, this supports that our developed HP-β-CD-g-poly(AA)/gelatin semi-IPN hydrogels are biocompatible in nature. The authors in [82] prepared and characterized pH-sensitive hydrogels based on poly(ethylene glycol) methyl ether, poly(ε-caprolactone), and IA. They did not observe any significant changes among the biochemical and hematological parameters of the control and treated groups.

#### 3.8.3. Histopathological Examination

Histopathology was carried out at the end of the study (the 14th day). All the animals were sacrificed and their vital organs were carefully removed. The weight of each organ was recorded, as shown in Table 8, and no significant weight changes were observed between the control and treated groups.

**Figure 14:**
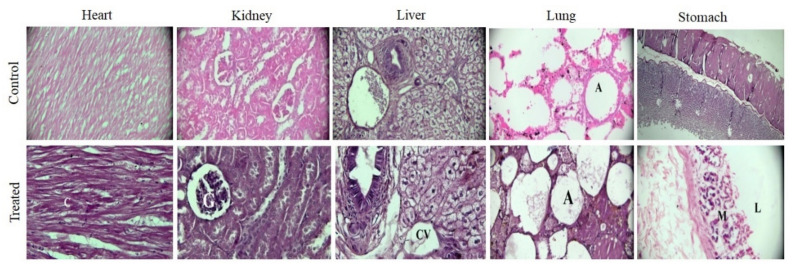
Histopathological images of control and treated animals. Accu-Scope 3001 microscope, fitted with 5-megapixel camera and 40× lens was used to take images after eosin and hematoxylin stains. Cardiomyocytes of heart (C), glomerulus (G), central vein (CV), alveolar sacs (A), lumen (L), and mucosa (M).

Cardiac muscles were present in normal shape with branched striated myofibers with nuclei in the center, thus reflecting non-toxicity to cardiac tissues. Kidneys appeared normal, with well-defined Bowman’s capsule around the glomerulus in control and treated groups. The liver showed a central vein and hepatocytes were arranged around it. No abnormality was observed in lung tissues. Alveolar sacs were present in both groups and no alveolar or bronchiolar collapse was observed. Stomach tissues of both groups showed normal gastric mucosa without any signs of ulceration or bleeding, as shown in Figure 14.

Similar findings were observed by [8], who conducted toxicity testing on pH-sensitive semi-IPN hydrogels based on chitosan, AA, and AMPS. They did not observe any histological changes in the treated group. Conclusively, these histological images confirm the nontoxic nature of the prepared gels and they are thus deemed suitable for in vivo applications.

## 4. Conclusions

The focus of this study was to formulate safe, biocompatible, pH-sensitive, and mucoadhesive semi-IPN hydrogels for the targeted delivery of DSP. We successfully developed semi-IPN hydrogels of HP-β-CD-g-poly(AA)/gelatin by using APS and MBA as initiators and crosslinkers, respectively. The swelling ratio decreased with a decreasing pH and vice versa. The optimized formulation (AG-1) was selected after carefully analyzing swelling, mucoadhesive, and sol–gel results of fifteen formulations developed by varying AA (16.66% to 83.33% *w*/*w*) and gelatin (0.33% to 2.66% *w*/*w*) concentrations. FTIR analysis hinted at the formation of the semi-IPN network while a rough surface with adhered drug particles were confirmed by SEM analysis. In vitro release study of the hydrogels presented a pH-dependent release from the gels by a non-Fickian mechanism. Detailed hemocompatibility and toxicity testing revealed the non-toxic nature of the prepared HP-β-CD-g-poly(AA)/gelatin, thus deeming it safe for biological systems.

## Figures and Tables

**Table 1:** Ingredients of HP-β-CD-g-poly(AA)/gelatin semi-IPN hydrogel formulations.

Code	AA g/100 g	Gelatin g/100 g	Code	AA g/100 g	Gelatin g/100 g	Code	AA g/100 g	Gelatin g/100 g
AG-1	16.66	0.33	AG-6	16.66	1.33	AG-11	16.66	2.66
AG-2	33.33		AG-7	33.33		AG-12	33.33	
AG-3	50		AG-8	50		AG-13	50	
AG-4	66.66		AG-9	66.66		AG-14	66.66	
AG-5	83.33		AG-10	83.33		AG-15	83.33	

HP-β-CD remains constant at 0.33 g/100 g while APS and MBA were 0.16 g/100 g and 0.33 g/100 g, respectively, in all formulations.

**Table 2:** Sol and gel (%) of HP-β-CD-g-poly (AA)/gelatin semi-IPN hydrogels.

Code	Gel (%)	Sol (%)	Code	Gel (%)	Sol (%)	Code	Gel (%)	Sol (%)
AG-1	90	10	AG-6	89.47	10.52	AG-11	87.09	12.90
AG-2	91.42	8.57	AG-7	90.62	9.37	AG-12	89.09	10.90
AG-3	92.42	7.57	AG-8	91.78	8.21	AG-13	90.58	9.41
AG-4	94.28	5.71	AG-9	92.78	7.21	AG-14	90.62	9.37
AG-5	95.68	4.31	AG-10	93.05	6.94	AG-15	91.26	8.73

**Table 3:** DSP release kinetics from AG-1 using Korsmeyer–Peppas model.

Model	Parameter	Value
Korsmeyer–Peppas	Correlation coefficient (R^2^)	0.9810
	Diffusion exponent (*n*)	0.453
	Release rate constant (K)	14.270

**Table 4:** Biochemical blood analysis.

Observations	Control Blood	Blood after Incubation	Blood with Blank Gels	Blood with Loaded Gels
Hemoglobin (10–15 g/dL)	13.63 ± 0.05	13.66 ± 0.11	13.13 ± 0.25	13.2 ± 0.1
P.V.C Hematocrit (%)	39.66 ± 1.52	37.36 ± 0.85	37.63 ± 0.58	38 ± 1
Total red blood cells (4.2–5.9 × 1012 L^−1^)	7.68 ± 0.38	7.53 ± 0.31	7.3 ± 0.07	7.43 ± 0.15
Mean corpuscular volume (80–96 fL)	53.2 ± 0.85	53.1 ± 0.60	53.33 ± 1.52	53 ± 1
Mean corpuscular hemoglobin (MCHb) (27–32 pg)	21.03 ± 2.98	19.4 ± 0.55	18.1 ± 1.15	17.13 ± 1.02
MCHb Concentration (g/dL)	36.76 ± 1.34	39.96 ± 0.70	37.6 ± 0.4	35.1 ± 2.85
Total white blood cells /cumm	8800 ± 100	8866 ± 152	8800 ± 264	9100 ± 200
Neutrophils (%)	46 ± 1	48 ± 1	43.66 ± 2.08	44 ± 2.64
Lymphocytes (%)	38.66 ± 1.52	35.66 ± 1.52	39.33 ± 2.08	41 ± 2.64
Monocytes (%)	12.66 ± 1.52	8 ± 1	10.33 ± 2.08	12.33 ± 1.52
Eosinophils (%)	6 ± 1	6.33 ± 1.52	6.66 ± 0.57	5.33 ± 1.52
Platelets count/cumm	248,666 ± 5131	308,000 ± 7000	317,666 ± 20,404	315,666 ± 2516

**Table 5:** Clinical examination of control and treated groups (*n* = 3).

Observations	Control (Group I)	Treated (Group II)
Mortality rate	Zero	Zero
Ocular toxicity (Lacrimation, redness of conjunctiva)	No toxicity	No toxicity
Dermal toxicity (Erythema, swelling, wound formation)	No toxicity	No toxicity
Illness signs (Loss of activity)	No sign	No sign
**Weight of Rabbits (kg)**		
Pretreatment	1.66 ± 0.25	1.73 ± 0.25
First Day	1.5 ± 0.17	1.6 ± 0.20
Seventh Day	1.36 ± 0.11	1.43 ± 0.24
Fourteen Day	1.46 ± 0.15	1.58 ± 0.10
**Water Intake (mL)**		
Pretreatment	184 ± 4.72	192 ± 4.93
First Day	182 ± 5.50	186 ± 3.21
Seventh Day	187 ± 4.93	189 ± 3.05
Fourteen Day	188 ± 1.52	190 ± 5.68
**Food Intake (g)**		
Pretreatment	68.33 ± 3.21	60.33 ± 3.51
First Day	69.33 ± 0.57	60 ± 3.00
Seventh Day	68.33 ± 2.51	65.33 ± 3.05
Fourteen Day	68.66 ± 1.52	65.66 ± 4.16

**Table 6:** Biochemical blood analysis.

Hematology	Control (Group I)	Treated (Group II)
Hemoglobin (10–15 g/dL)	13.7 ± 1.21	13.06 ± 0.80
TLC (4.5–11 × 109 L^−1^)	4.3 ± 0.81	5 ± 1
Red Blood Cells (4.2–5.9 × 1012 L^−1^)	5.49 ± 0.62	5.65 ± 0.35
Platelets (150–400 × 109 L^−1^)	166 ± 11.15	186 ± 38.21
Monocytes (2–8%)	5.33 ± 1.52	4.33 ± 2.30
Neutrophils (40–60%)	55 ± 4.35	41 ± 17.52
Lymphocytes (20–40%)	75.33 ± 4.61	76.66 ± 4.16
Eosinophils (1–4%)	4 ± 1	3.33 ± 1.52
Mean Corpuscular Volume (80–96 fL)	65.43 ± 3.98	70.2 ± 7.70
MCHb (27–32 pg)	25.16 ± 4.18	25.6 ± 2.95
MCHb Concentration (32–36%)	33.33 ± 2.89	35.46 ± 5.62

**Table 7:** Lipid, kidney, and liver function test.

Biochemical Analysis	Control (Group I)	Treated (Group II)
**Lipid Profile**		
Cholesterol (10–80 mg/dL)	63.93 ± 3.45	65.33 ± 7.23
Triglycerides (46–68 mg/dL)	54.28 ± 1.82	58.33 ± 8.02
**Renal Profile**		
Creatinine (0.2–0.9 mg/dL)	0.83 ± 0.20	0.7 ± 0.1
Urea (10–50 mg/dL)	30 ± 10	24.66 ± 13.61
Uric acid (3.4–7.1 mg/dL)	5.13 ± 1.09	5.56 ± 0.51
**Liver Profile**		
Alanine aminotransferase (ALT) (17–77 IU/L)	34.66 ± 5.03	32 ± 11.13
Aspartate aminotransferase (AST) (54–298 IU/L)	25.33 ± 11.71	21 ± 10.41

**Table 8:** Vital organs weight difference between both groups (*n* = 3).

Group	Stomach (g)	Kidney (g)	Heart (g)	Liver (g)	Lung (g)
**Control**	12.33 ± 1.85	10.69 ± 1.16	4.08 ± 0.62	34.09 ± 3.57	16.31 ± 3.05
**Treated**	14.66 ± 3.51	8.34 ± 1.47	3.46 ± 0.47	44.15 ± 7.93	13.33 ± 4.16

## Data Availability

Most of the data is presented in main article. Raw or processed data required to reproduce these findings cannot be shared at this time due to technical or time limitations.
